# Obesity and acute type A aortic dissection: unraveling surgical outcomes through the lens of the upper hemisternotomy approach

**DOI:** 10.3389/fcvm.2024.1301895

**Published:** 2024-02-01

**Authors:** Lin Xia, Yu Liu, Zhonglu Yang, Yuguang Ge, Lu Wang, Yejun Du, Hui Jiang

**Affiliations:** Department of Cardiovascular Surgery, General Hospital of Northern Theater Command, Shenyang, Liaoning, China

**Keywords:** acute type A aortic dissection, upper hemisternotomy, body mass index, postoperative hypoxemia, cardiovascular surgery outcomes

## Abstract

**Background:**

Acute type A aortic dissection (ATAAD) is a pressing cardiovascular emergency necessitating prompt surgical intervention. Obesity, a pervasive health concern, has been identified as a significant risk factor for ATAAD, introducing unique surgical challenges that can influence postoperative outcomes. This study aimed to investigate the outcomes of ATAAD surgery across various body mass index (BMI) categories, focusing on the implications of the upper hemisternotomy (UHS) approach.

**Methods:**

Between April 2017 and October 2023, 229 patients diagnosed with ATAAD underwent aortic arch intervention via UHS at the General Hospital of Northern Theater Command. Based on BMI (WS/T 428-2013), patients were categorized into normal weight, overweight, and obese. The primary outcomes included perioperative parameters, intraoperative details, and postoperative complications, with specific emphasis on hypoxemia, defined by the Berlin criteria as a PaO2/FiO2 ratio of ≤300 mmHg.

**Results:**

The average age of the cohort was 50.1 ± 11.2 years with a male predominance (174 males). Preoperatively, 49.0% presented with hypoxemia, with the Obese group exhibiting a significantly elevated rate (77.9%, *P* < 0.001). Postoperatively, while the Normal group demonstrated a lower thoracic drainage volume 24 h post-surgery [180.0 (140.0) ml; *P* < 0.001], the Obese group indicated prolonged durations for mechanical ventilation and ICU stay, without statistical significance. Unlike the Normal and Overweight groups, the Obese group showed no notable changes in pre- and postoperative PaO2/FiO2 ratio. No significant difference was observed in severe postoperative complications among the groups. Further ROC curve analysis identifies a BMI cutoff of 25.5 for predicting postoperative hypoxemia, with 76.3% sensitivity and 84.4% specificity. And multivariate analysis reveals BMI and preoperative hypoxemia as independent predictors of postoperative hypoxemia.

**Conclusion:**

Obesity, although presenting unique challenges in ATAAD interventions, does not necessarily portend adverse outcomes when managed with meticulous surgical planning and postoperative care. The study emphasizes the significance of individualized patient assessment and tailoring surgical strategies, suggesting the potential of UHS in addressing the surgical intricacies posed by obesity in ATAAD patients. Further research is warranted to consolidate these findings.

## Introduction

Acute type A aortic dissection (ATAAD) is a life-threatening medical emergency, arising from an intimal tear within the aortic wall. This demands immediate intervention to avert catastrophic outcomes (1). In the contemporary health landscape, obesity has emerged as a salient risk factor for a plethora of cardiovascular ailments, ATAAD being no exception (2). This correlation can be attributed to various pathophysiological mechanisms induced by obesity, such as hemodynamic stress, hypertension, atherosclerosis, metabolic dysregulation, and compromised tissue repair (3). Importantly, obese patients diagnosed with ATAAD introduce a unique set of surgical intricacies. Challenges encompassing surgical access, technical nuances, and considerations pertinent to cardiopulmonary bypass (CPB) and postoperative management necessitate a specialized approach for this demographic (4). It becomes imperative for surgeons to recognize and navigate these intricacies to ensure optimal postoperative outcomes.

Upper hemisternotomy (UHS) offers an array of advantages for patients undergoing aortic surgical interventions. These encompass superior exposure for supra-cardiac structures, minimized surgical trauma, reduced hemorrhage, aesthetic benefits, diminished sternal complications, and expedited patient recovery (5, 6). Contrasted with the traditional full sternotomy, the UHS technique is characterized by a more conservative incision and limited lung manipulation, culminating in attenuated pulmonary trauma and preservation of pulmonary function (7, 8). This, in theory, diminishes the postoperative risk of hypoxemia. Our prior research underscored the application of UHS in total arch replacement (TAR) (9). Nevertheless, it's pivotal to recognize that the prognostic implications of UHS can be heterogeneous, contingent upon individualized patient parameters. For instance, the obese subset might still grapple with an accentuated risk of postoperative hypoxemia, attributed to factors such as diminished pulmonary volume and heightened respiratory workload (10). Thus, exploring TAR outcomes via UHS across varying body mass index (BMI) strata is paramount, intending to refine surgical methodologies through the adoption of minimally invasive paradigms.

## Methods

### Patient population

Approval for this study was granted by the Institutional Ethic Research Board (Protocol #K-2020019). All participants were thoroughly informed about the study's aims and procedures, with explicit consent obtained. From April 2017 and October 2023, a total of 229 patients diagnosed with definitive ATAAD based on computed tomographic arteriography (CTA) underwent aortic arch intervention with mild hypothermic circulatory arrest (MiHCA) via the upper hemisternotomy (UHS) approach at the General Hospital of Northern Theater Command. The study included interventions involving TAR and frozen elephant trunk (FET) procedures performed under mild hypothermic circulatory arrest via the UHS approach. Exclusion criteria encompassed preoperative neurologic complications, abdominal complications (e.g., acute hepatic failure, gastrointestinal bleeding, and acute renal failure), and concomitant procedures requiring full sternotomy (e.g., coronary heart disease, mitral valve disease, and congenital heart disease). BMI values were determined upon admission by calculating the weight-to-height squared ratio (kg/m^2^). According to the Chinese criterion (WS/T 428-2013), patients were categorized into three groups based on their BMI at the time of surgery: normal weight (18.5 ≤ BMI < 24 kg/m^2^), overweight (24 ≤ BMI < 28 kg/m^2^), and obese (BMI ≥ 28 kg/m^2^). Arterial blood gas analysis was conducted to calculate the arterial partial pressure of oxygen to fraction inspired oxygen (PaO2/FiO2) during the perioperative period. Based on the Berlin criteria, hypoxemia is defined as a PaO2/FiO2 ratio of ≤300 mmHg (11).

### Surgical procedure

**Figure 1:**
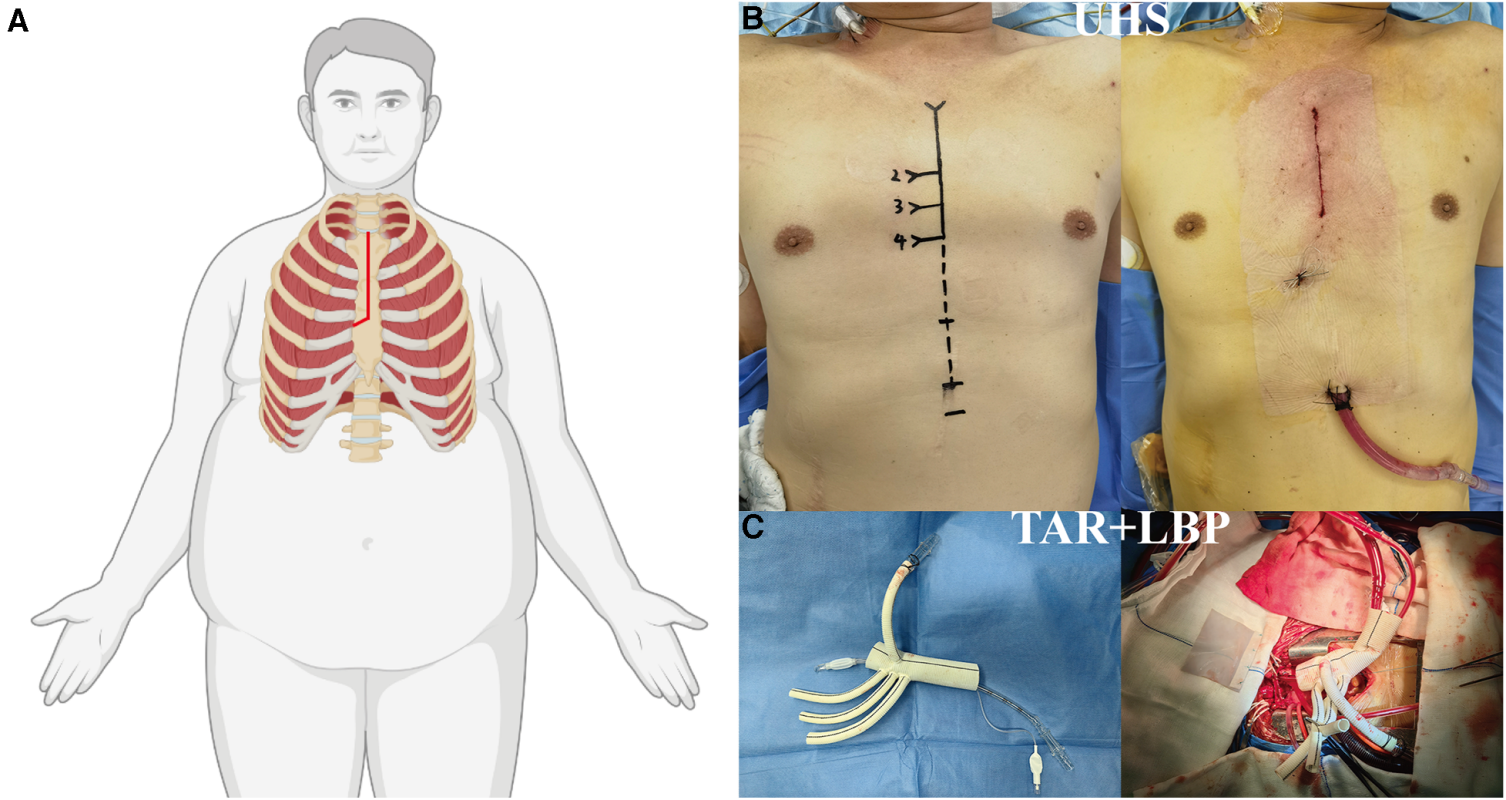
Surgical implications of obesity in ATAAD managed via UHS. (**A**) Illustration of the “J”-shaped incision technique optimized for the obese patient undergoing UHS. (**B**) Contrast between preoperative and postoperative incisions. (**C**) Depictions of TAR and LBP procedures. ATAAD, acute type A aortic dissection; UHS, upper hemisternotomy; TAR, total arch replacement; LBP, lower body perfusion.

A longitudinal incision was made from the sternal notch to the fourth intercostal space, which was further extended to the right fourth intercostal space. CPB was established by cannulating the direct innominate artery (right subclavian artery, right or left carotid artery) for arterial access and by directly cannulating the right atrium for venous access. A left ventricular vent was inserted through the right superior pulmonary vein. Antegrade cardioplegia was administered through coronary orifices following aortotomy. MiHCA was initiated when the nasopharyngeal temperature reached 30°C–32°C. Subsequently, the proximal ends of the left subclavian artery, left common carotid artery, and innominate artery were sequentially closed using Hem-o-lok ligating clips. Bilateral selective antegrade cerebral perfusion (bSACP) was initiated through the arterial cannulation, with a 15Fr femoral arterial cannula placed in the distal end of the left common carotid artery after cross-clamping of the innominate artery, allowing perfusion of the brain using the cardioplegia pump. Near-infrared reflection spectroscopy (NIRS) monitoring was employed for cerebral protection. Circulatory arrest was achieved after occluding the innominate artery, and frozen elephant trunk (FET) implantation was performed. Lower body perfusion (LBP) was initiated using a 16Fr cannula with an occlusion balloon (Longlaifu, Changzhou, China) inserted through the distal artery of the 4-branch prosthetic graft (Hemashield Platinum Double Velour Vascular Graft; MAQUET, La Ciotat, France), providing blood flow recovery to the lower body at a rate of 25 ml/kg·min. Once the distal aorta incorporating the stent graft was securely attached to the distal end of the 4-branch prosthetic graft, LBP was initiated via the perfusion limb of the 4-branch prosthetic graft. The sequence of anastomosis to the prosthetic graft included the left common carotid artery, proximal aortic stump, left subclavian artery, and innominate artery. Upon completion of the anastomosis to the left common carotid artery, CPB flow gradually returned to normal, and the process of rewarming commenced (Figure 1).

### Statistical analysis

Retrospective data collection and analysis were performed using SPSS Version 25.0 (SPSS Inc., Chicago, IL, USA). Continuous variables were presented as mean ± standard deviation or medians (Inter Quartile Range, IQR), while categorical variables were presented as frequency (%). The distribution of categorical variables between groups was compared using either a chi-squared test or Fisher's exact probability test. For continuous variables, statistical analysis was conducted using the One-Way ANOVA test with post-hoc LSD test, Welch test with post-hoc Games-Howell test, or Kruskal-Wallis *H* test with pairwise multiple comparisons. In cases where the data did not meet the assumptions of normality, the non-parametric Mann-Whitney *U* test was utilized for between-group comparisons. To determine the independent predictors of postoperative outcomes, binary Logistic regression analysis was conducted. A significance level of less than 0.05 was considered statistically significant.

## Results

### Baseline characteristics

In our cohort of 229 consecutive patients who satisfied the predefined inclusion and exclusion parameters, the average age was 50.1 ± 11.2 years. Of these, 174 were males, and 55 were females. The perioperative characteristics are detailed in Table 1. Notably, 49.8 of these patients demonstrated preoperative hypoxemia, with a PaO2/FiO2 ratio of ≤300 mmHg. Further subgroup analysis revealed that the Obese group had a significantly higher rate of preoperative hypoxemia (77.9) than the other two groups (*P* < 0.001). Among the participants, 50.7 reported a history of smoking, while 72.5 had a history of hypertension. No other clinically significant differences in the medical backgrounds were discernible among the three groups.

**Table 1:** Baseline characteristics.

Clinical variables	Total	Normal	Overweight	Obese	*P*
	(*n* = 229)	(*n* = 68)	(*n* = 84)	(*n* = 77)	
Age (Years)	50.1 ± 11.2	50.4 ± 10.6	50.1 ± 11.5	49.8 ± 11.6	.942^a^
Male, *n* (%)	174 (76.0)	49 (72.1)	66 (78.6)	59 (76.6)	.638^b^
BMI (kg/m^2^)	26.0 (5.3)	22.0 (3.3)	25.5 (2.0)**	29.8 (2.6)**^,††^	<.001^c^
LVEF (%)	58.0 (3.0)	58.0 (3.0)	58.0 (4.0)	58.0 (3.0)	.424^c^
Smoking, *n* (%)	116 (50.7)	38 (55.9)	37 (44.0)	41 (53.2)	.299^b^
Diabetes, *n* (%)	7 (3.1)	2 (2.9)	3 (3.6)	2 (2.6)	1.000^d^
Hypertension, *n* (%)	166 (72.5)	49 (72.1)	57 (67.9)	60 (77.9)	.359^b^
BAV, *n* (%)	3 (1.3)	1 (1.5)	1 (1.2)	1 (1.3)	1.000^d^
Marfan's syndrome, *n* (%)	6 (2.6)	2 (2.9)	0	4 (5.2)	.097^d^
PaO2/FiO2 (mmHg)	299.0 (127.5)	313.5 (48.2)	310.8 (67.4)	177.0 (125.6)**^,††^	<.001^c^
Hypoxemia, *n* (%)	114 (49.8)	19 (27.9)	35 (41.7)	60 (77.9)*^,†^	<.001^b^

BMI, body mass index; LVEF, Left ventricular ejection fraction; BAV, Bicuspid aortic valve.

^a^ One-Way ANOVA test with post-hoc LSD test.

^b^ chi-squared test.

^c^ Kruskal-Wallis *H* test with all pairwise multiple comparisons.

^d^ Fisher's exact probability test.

* *P* < .05.

** *P* < .001 vs. the Normal group.

^†^ *P* < .05.

^††^ *P* < .001 vs. the Overweight group.

### Intraoperative details

Table 2 delineates the specifics of the intraoperative parameters. Comparative analysis among the groups revealed no statistically significant disparities concerning CPB duration, aortic cross-clamp time, and circulatory arrest duration. The median minimum nasopharyngeal temperature during the procedure was recorded as 26.9°C. Notably, extracorporeal membrane oxygenation (ECMO) intervention was necessitated in three patients.

**Table 2:** Intraoperative details.

Clinical variables	Total	Normal	Overweight	Obese	*P*
	(*n* = 229)	(*n* = 68)	(*n* = 84)	(*n* = 77)	
CPB time (min)	160.0 (50.0)	154.5 (54.0)	157.5 (50.0)	163.0 (54.0)	.482^a^
Cross-clamp time (min)	91.0 (34.0)	90.0 (32.0)	90.0 (34.0)	97.0 (40.0)	.333^a^
Circulatory arrest time (min)	6.0 (5.0)	5.3 (2.0)	6.0 (6.0)	7.0 (5.0)	.346^a^
Minimum nasopharyngeal temperature (°C)	26.9 (2.0)	29.2 (1.0)	29.8 (2.0)	29.3 (1.0)	.054^a^
Combined procedure					
Commissural resuspension, *n* (%)	128 (55.9)	37 (54.4)	46 (54.8)	45 (58.4)	.858^b^
Bentall, *n* (%)	26 (11.4)	7 (10.3)	6 (7.1)	13 (16.9)	.143^b^
David, *n* (%)	5 (2.2)	0	4 (4.8)	1 (1.3)	.164^c^
TAR + FET, *n* (%)	229 (100.0)	68 (100.0)	84 (100.0)	77 (100.0)	
ECMO, *n* (%)	3 (1.3)	1 (1.5)	1 (1.2)	1 (1.3)	1.000^c^

CPB, Cardiopulmonary bypass; TAR, Total arch replacement; FET, Frozen elephant trunk; ECMO, Extracorporeal membrane oxygenation.

^a^ Kruskal-Wallis *H* test with all pairwise multiple comparisons.

^b^ Chi-squared test.

^c^ Fisher's exact probability test.

### Postoperative outcomes

**Figure 2:**
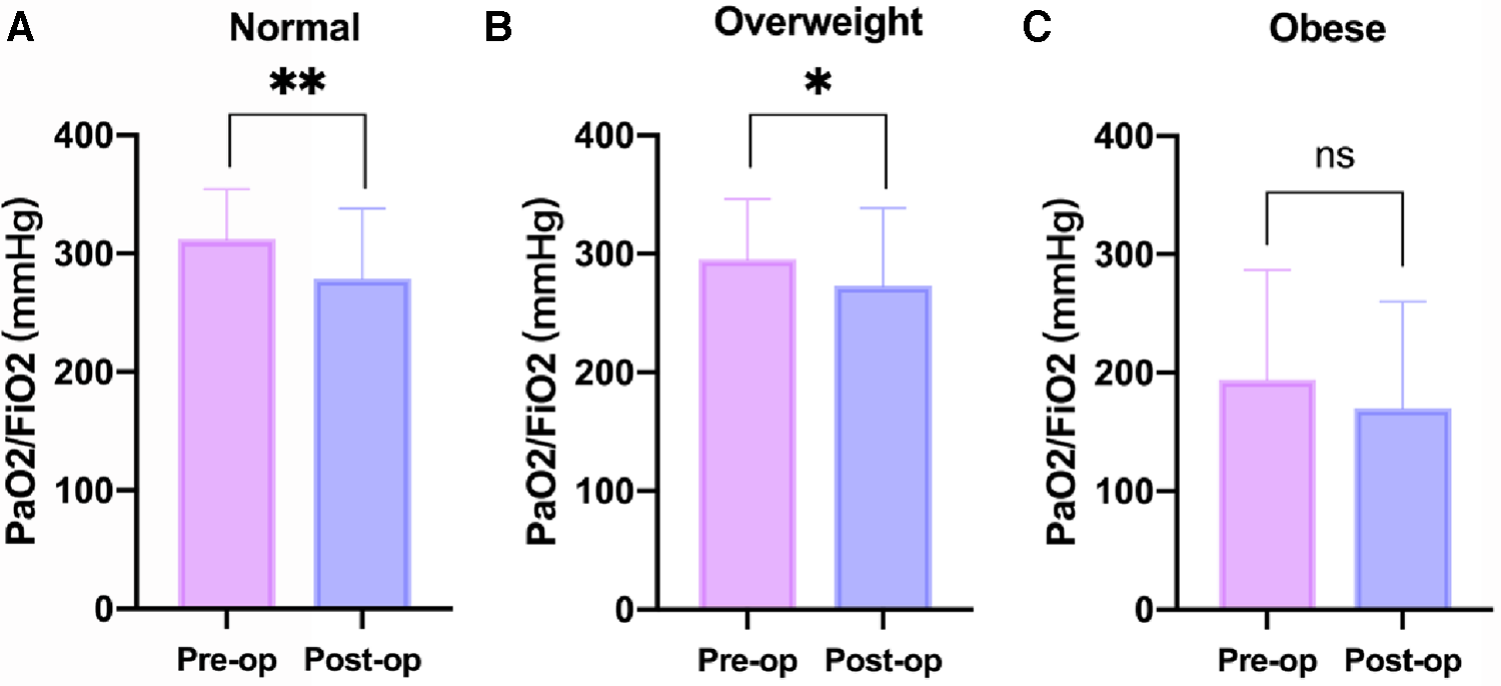
Changes in PaO2/FiO2 before and after operation at different BMI levels. (**A**) Normal Group (18.5 ≤ BMI < 24 kg/m^2^). (**B**) Overweight Group (24 ≤ BMI < 28 kg/m^2^). (**C**) Obese Group (BMI ≥ 28 kg/m^2^). BMI, body mass index; Pre-op, preoperative; Post-op, Postoperative. *P < .05, **P < .01 vs. the Pre-op group.

Table 3 provides a comprehensive overview of the postoperative outcomes across the three groups. When examining the thoracic drainage volume 24 h post-surgery, the Normal group demonstrated a significantly reduced mean volume [180.0 (140.0) ml] compared to the other groups [250.0 (180.0) ml and 290.0 (220.0) ml; *P* = 0.001]. Although the Obese group exhibited prolonged durations for both postoperative mechanical ventilation and ICU stay, the differences weren't statistically significant compared to the other groups. Analyzing postoperative complications, a marked increase in the incidence of postoperative hypothermia was observed in the Obese group (83.1%), which was notably higher than the rates in the other two groups (45.6% and 52.4; *P* = <.001). In contrast to the Normal and Overweight groups, the Obese group exhibited no significant alterations in the PaO2/FiO2 ratio when comparing preoperative and postoperative levels (Figure 2). Further, there was no significant discrepancy in the occurrence rates of complications such as reintubation, re-exploration due to bleeding, stroke, and continuous renal replacement therapy (CRRT) among the three groups.

**Table 3:** Postoperative outcomes.

Clinical variables	Total	Normal	Overweight	Obese	*P*
	(*n* = 229)	(*n* = 68)	(*n* = 84)	(*n* = 77)	
Ventilation time (h)	32.0 (48.1)	31.0 (70.2)	23.0 (50.2)	39.4 (43.8)	.706^a^
ICU stay (h)	56.8 (69.4)	44.5 (25.1)	45.5 (81.5)	62.2 (47.0)	.900^a^
In-hospital stay (d)	14.0 (9.0)	13.0 (9.0)	15.0 (11.0)	14.0 (8.0)	.569^a^
First 24 h chest tube drainage (ml)	240.0 (200.0)	180.0 (140.0)	250.0 (180.0)*	290.0 (220.0)**	.001^a^
Transfusion, *n* (%)	155 (68.0)	49 (73.1)	56 (66.7)	50 (64.9)	.545^b^
PaO2/FiO2 (mmHg)	246.1 (137.3)	302.6 (92.0)	261.0 (109.0)	159.0 (136.0)***^,††^	<.001^a^
Hypoxemia, *n* (%)	139 (60.7)	31 (45.6)	44 (52.4)	64 (83.1)*^,†^	<.001^b^
Reintubation, *n* (%)	10 (4.4)	4 (6.0)	2 (2.4)	4 (5.2)	.511^c^
Re-exploration for bleeding, *n* (%)	5 (2.2)	2 (3.0)	1 (1.2)	2 (2.6)	.740^c^
Stroke, *n* (%)	17 (7.5)	8 (11.9)	4 (4.8)	5 (6.5)	.271^c^
CRRT, *n* (%)	30 (13.2)	9 (13.4)	10 (11.9)	11 (14.3)	.902^b^
In-hospital mortality, *n* (%)	18 (7.9)	7 (10.3)	4 (4.8)	7 (9.1)	.400^b^

ICU, intensive care unit; CRRT, continuous renal replacement therapy.

^a^ Kruskal-Wallis *H* test with all pairwise multiple comparisons.

^b^ Chi-squared test.

^c^ Fisher's exact probability test.

* *P* < .05.

** *P* < .01.

*** *P* < .001 vs. the Normal group.

^†^ *P* < .05.

^††^ *P* < .001 vs. the Overweight group.

### Multivariate regression and ROC curve analysis

**Figure 3:**
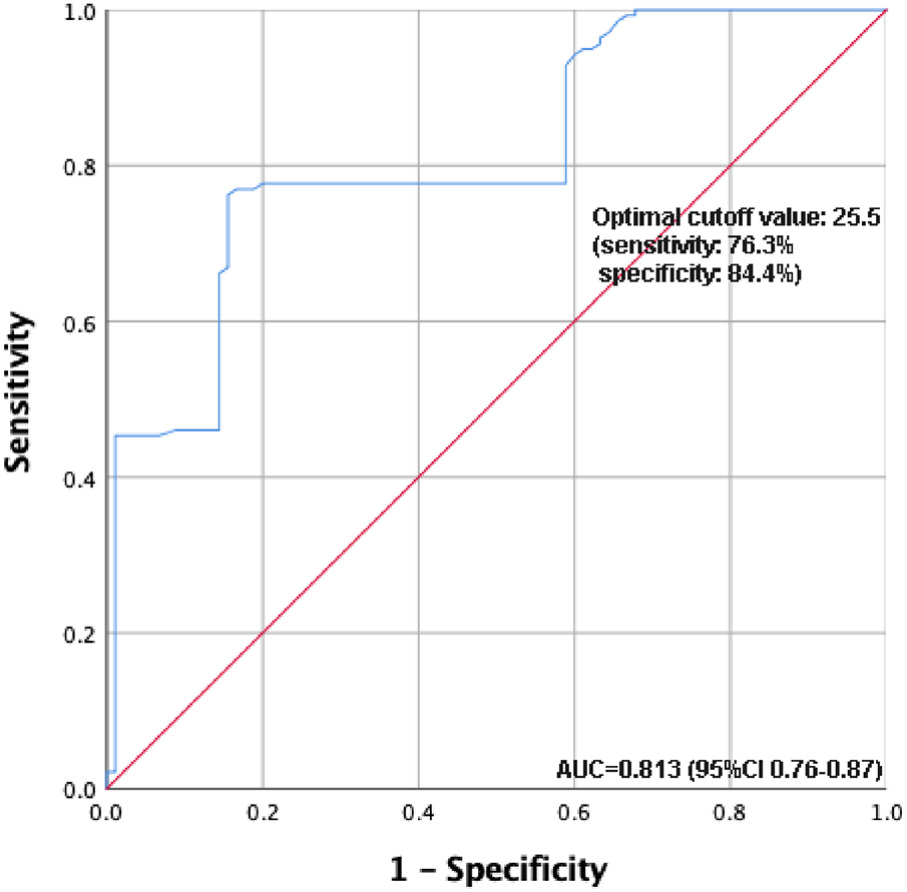
ROC curve analysis of the predictive value of BMI for postoperative hypoxemia. ROC, receiver operating characteristic; BMI, body mass index.

Table 4 presents the results of the multivariate regression analysis. Clinical variables from the preoperative and intraoperative phases were initially subjected to univariate regression analysis. Statistically significant factors, including BMI, the preoperative PaO2/FiO2 ratio, and preoperative hypoxemia, were incorporated into the multivariate model. The results indicate that BMI and preoperative hypoxemia are independent risk factors for postoperative hypoxemia. Furthermore, based on the ROC curve analysis evaluating BMI's predictive power for postoperative hypoxemia, the optimal cutoff value is determined to be 25.5, with a sensitivity of 76.3% and a specificity of 84.4% (Figure 3).

**Table 4:** Multivariate analysis of independent risk factors for postoperative hypoxemia.

Clinical variables	Coefficient	Standard error	OR (95% CI)	*P*
BMI (kg/m^2^)	0.216	0.075	1.241 (1.072–1.436)	.004
PaO2/FiO2 (mmHg)	0.004	0.006	1.004 (0.992–1.016)	.511
Preoperative Hypoxemia, *n* (%)	4.188	0.917	65.896 (10.925–397.473)	<.001

BMI, body mass index.

## Discussion

Acute type A aortic dissection (ATAAD) is an emergent and critical cardiovascular pathology requiring timely intervention, and obesity has been underscored as a pertinent risk factor, introducing surgical complexities that can potentially influence outcomes (12, 13). Our study, which investigated the postoperative consequences across different BMI strata following TAR via UHS, provided crucial insights into the interplay between obesity and surgical intricacies in ATAAD.

One of the pivotal findings of our investigation pertained to the notable variance in preoperative hypoxemia across BMI categories. The Obese group manifested a significantly elevated rate of preoperative hypoxemia (74.0%), a statistic surpassing that of its counterparts. This can be rooted in the physiopathological modifications synonymous with obesity, such as reduced pulmonary volume and augmented respiratory effort (14). To address this, we propose preoperative optimization strategies for respiratory function, including preoperative pulmonary rehabilitation or noninvasive ventilation support, particularly for obese patients.

The application of UHS has traditionally been championed for its minimally invasive nature, which subsequently offers a plethora of advantages ranging from improved exposure to reduced surgical trauma (15, 16). Our prior endeavors have resonated with these merits, elucidating the effectiveness of UHS in TAR (9, 17, 18). However, our current study accentuates the necessity to delve deeper, understanding that a one-size-fits-all strategy might not be pertinent, especially in the diverse landscape of patient physiologies. Anticipatory management plans, such as tailored perioperative monitoring and aggressive postoperative physiotherapy, may be beneficial for obese patients to reduce the duration of mechanical ventilation and ICU stays (19). The findings from our postoperative data accentuate this further. While the Normal group illustrated a significantly reduced thoracic drainage volume 24 h post-surgery, the Obese group, even with the conservative approach of UHS, faced prolonged mechanical ventilation and ICU stay durations, albeit these did not attain statistical significance. Enhanced recovery protocols, including vigilant fluid management, could further improve postoperative outcomes for these patients (20).

It's intriguing to observe that despite the aforementioned challenges, the Obese group demonstrated a lesser post-surgery rise in patients exhibiting hypoxemia compared to their preoperative state, especially when set against the Normal groups. This possibly hints at the potential adaptive physiological mechanisms in the obese cohort, which might be more resilient to certain intraoperative and postoperative challenges.

Furthermore, despite the unique challenges posed by obesity, our study did not find a significant difference in severe postoperative complications, such as reintubation, re-exploration due to bleeding, stroke, and the need for continuous renal replacement therapy (CRRT) among the groups. This underscores the efficacy of a surgical approach and postoperative management that are tailored to the individual patient's needs. For example, employing wound care protocols designed for patients with diminished tissue repair mechanisms, as well as personalized postoperative nutritional plans to promote recovery. This underlines the efficacy of the surgical approach and postoperative management, suggesting that while obesity presents certain complexities, they can be adeptly managed with the right surgical strategy and postoperative care (21, 22).

### Limitation

While our study offers valuable preliminary insights into the impact of obesity on surgical outcomes in ATAAD, we recognize its scope as an initial explorative step. The retrospective nature of our research poses inherent limitations, including the possibility of unrecognized biases. Our single-center experience provides a foundation for hypothesis generation rather than definitive conclusions. Thus, we emphasize that our findings serve as a “first interesting impression” that requires corroboration through large-scale, multi-center studies designed to mitigate potential confounders. Such studies would not only confirm our observations but also enhance the generalizability of the results, providing a clearer delineation of obesity's role in the management and prognosis of ATAAD.

To substantiate and build upon the patterns observed in our study, future research endeavors should aim to include a broader patient population, potentially revealing variances across different demographics and healthcare settings. We hope that our study will serve as a catalyst for subsequent research, prompting further examination of tailored surgical approaches for obese patients in the context of ATAAD.

## Conclusion

In conclusion, while obesity undeniably introduces an additional layer of complexity in ATAAD interventions, with meticulous surgical planning and postoperative care, commendable outcomes can still be achieved. Our study reiterates the significance of individualized patient assessment, ensuring that surgical strategies are tailored to meet the unique demands presented by varying patient demographics and physiological states.

## Data Availability

The raw data supporting the conclusions of this article will be made available by the authors, without undue reservation.
